# Solvent Vapour Detection with Cholesteric Liquid Crystals—Optical and Mass-Sensitive Evaluation of the Sensor Mechanism^†^

**DOI:** 10.3390/s100504887

**Published:** 2010-05-12

**Authors:** Adnan Mujahid, Helen Stathopulos, Peter A. Lieberzeit, Franz L. Dickert

**Affiliations:** Department of Analytical Chemistry, University of Vienna, Waehringer Strasse 38, A-1090 Vienna, Austria; E-Mails: adnan.mujahid@univie.ac.at (A.M.); helen.stathopulos@univie.ac.at (H.S.); peter.lieberzeit@univie.ac.at (P.A.L.)

**Keywords:** cholesteric liquid crystals, organic solvent vapours, pitch, optical absorbance, QCM

## Abstract

Cholesteric liquid crystals (CLCs) are used as sensitive coatings for the detection of organic solvent vapours for both polar and non-polar substances. The incorporation of different analyte vapours in the CLC layers disturbs the pitch length which changes the optical properties, *i.e.*, shifting the absorption band. The engulfing of CLCs around non-polar solvent vapours such as tetrahedrofuran (THF), chloroform and tetrachloroethylene is favoured in comparison to polar ones, *i.e.*, methanol and ethanol. Increasing solvent vapour concentrations shift the absorbance maximum to smaller wavelengths, e.g., as observed for THF. Additionally, CLCs have been coated on acoustic devices such as the quartz crystal microbalance (QCM) to measure the frequency shift of analyte samples at similar concentration levels. The mass effect for tetrachloroethylene was about six times higher than chloroform. Thus, optical response can be correlated with intercalation in accordance to mass detection. The mechanical stability was gained by combining CLCs with imprinted polymers. Therefore, pre-concentration of solvent vapours was performed leading to an additional selectivity.

## 1. Introduction

Monitoring of hazardous organic vapours is of great importance in different laboratories, industries and congested public places. The detection becomes more crucial for those personnel who are continuously working with or exposed to these chemicals in chemical plants as these vapours may have severe health effects. The flammable nature of organic vapours can lead to the formation of explosive mixtures with air, which is another serious issue which needs to be considered. The levels of these vapours are predefined by environmental protection authorities in order to ensure public health and safety. Already established methods for their detection are mostly based on electrochemical methods, electronic noses and optical sensors. Electrochemical [1,2] detection systems measure the change in the conductivity on metal oxide surface upon interaction with organic vapours. Optical sensors [3–5] have a much wider range of techniques available for organic solvent vapour detection that includes fluorescence, spectroscopy imaging, optical fibers, near infra red (NIR) laser diodes, surface plasmon resonance (SPR) and others. The sensitive materials used in these devices are vapochromic materials [6], thin co-ordination polymer films [7], metal based phthalocyanines [8], fluoroclathrands [9] and liquid crystals [10–13].

In all these sensing materials, cholesteric liquid crystals (CLCs) [14–19] provide a different sensing approach for polar solvent vapours in comparison to others, particularly for non polar ones that possesses low reactivity for chemical interactions. CLCs are also referred to as chiral nematic liquid crystals because they form helical patterned layers over each other that exhibit chirality. There is a certain periodic variation in the patterning of these helical layers which is recognized as pitch of CLCs, *i.e.*, equal to the distance covered to make a complete rotation of 360°. The incorporation of analyte vapours in CLCs helical layers tends to change the pitch and that ultimately alter the optical properties. This feature of CLCs could prove beneficial for organic vapour detection as the incorporation of analyte molecules changes the anisotropic phase of CLC. The nature of the interaction between analyte vapours and CLCs is attributed to short range attractive forces similar to those present between helical layers. The application of CLCs [20] for aromatic and halogenated solvent vapours sensing by using mixture of cholesteric esters has already been reported. The suitable selection of different cholesteric esters combinations had proven very effective to tune the optical properties of sensing material. These studies also provide the information about humidity effect and temperature dependence on absorbance of organic vapours detection by CLCs.

Winterbottom *et al.* [21] had also employed a similar approach for the detection of organic vapours in air using a mixture of cholesteryl nonanoate and cholesteryl chloride. Interestingly, they had measured the reflectance of CLCs in contrast to a previous method where absorbance was the sensing parameter. The sensitivity and selectivity of designed CLCs could be used to analyze different vapour mixtures samples in air by the multivariate data analysis technique.

The present work focuses on applications of CLCs, *i.e*., chiral biphenyls, for sensing of polar and non-polar solvent vapours having less pronounced reactivity. The effect of increasing concentration of solvent vapours on λ_max_ has been studied. The incorporation of volatile organic compounds in CLCs leads to alteration of their optical properties which were recorded by spectrophotometer and plotted against their molar mass. In parallel the CLCs have been coated on mass sensitive devices such as QCMs and the normalized mass effect was compared to the optical absorbance of analyte vapours. The variation in the results of two different transducing systems, *i.e.*, optical and mass sensitive techniques using the same sensitive coating material for the very same analyte has been investigated. In this way steric effects and pure mass effects can be compared. In both sensing schemes the incorporation of analyte vapours in CLCs was done physically, which have no bleaching effect, in contrast to dyes. To improve the mechanical stability with respect to flooding the sensor layer and the sensitivity of coating material, CLCs have been integrated in a matrix of imprinted polymers. The difference between the pure cholesteric liquid crystals and in a polymer matrix was evaluated to compare selectivity. Moreover, the optimal amount of LCs for imprinted polymer was also investigated to achieve the finest sensing system. The overall comparison of optical methods with mass sensitive technique offers an inside view of the changes in the pitch length and twisting angle of CLCs layers, which are very attractive for studying the morphological structure of analyte vapours.

## 2. Experimental Section

### 2.1. Chemicals and Reagents

All the chemicals and reagents were purchased as the highest available purity of analytical grade and used without any prior treatment. Divinylbenzene, styrene, azobisisobutyronitrile (AIBN) and tetrahydrofuran were used for polymer synthesis. The analytes tested with cholesteric liquid crystals were e.g., methanol, ethanol, tetrahydrofuran, chloroform and tetrachloroethylene. CLCs (Licritherm TM 1013) were obtained from Merck.

### 2.2. Synthesis of Molecularly Imprinted Layer

A mixture of styrene monomers along with 30% of divinylbenzene by weight as cross linker was polymerized in an 80-fold excess of solvent. The reaction was initiated by AIBN which was used in 1% by weight of total contents. For imprinting cholesteric liquid crystals (TM1013) were added to above mixture in a small fraction. As the polymerization is photoinduced the coated MIP layers were exposed to UV light at λ = 365 nm for 10 minutes which hardened the layer coatings. The layer height was calculated to be 1.33 μm.

### 2.3. Gas Mixing Apparatus

For producing analyte vapours, a temperature controlled gas mixing apparatus under defined humidity conditions was designed. A pressurized air stream of approximately 2 bar was purified from oil traces and passed through silica gel to remove moisture contents. Tylan FC 2900 air flow controllers were used in this experiment to regulate the amount of air within the desired range. The dry gas was passed through a water bottle to produce a defined humidity level and then mixed with solvent vapours. This defined humidity level was controlled by an integrated Rotronic Y A-100C humidity sensor. As CLCs are highly temperature sensitive the temperature of the whole gas mixing apparatus and the measurement chamber is controlled by a thermostat. This allows smooth functioning without facing any temperature fluctuations.

### 2.4. Measurements Devices

The UV/VIS measurements were performed on a Perkin Elmer Lambda 12 spectrophotometer (resolution 2 nm). The infrared spectra were recorded on a Perkin Elmer 2000 FTIR spectrometer with a ZnSe ATR equipment. A 10 MHz AT-cut QCM has been used for performing mass sensitive measurements.

## 3. Results and Discussion

**Figure 1.**
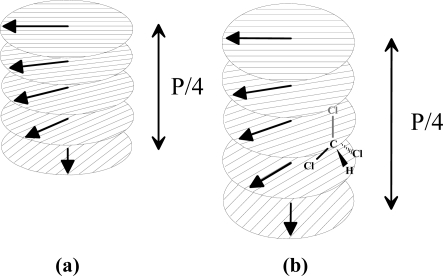
Representation of cholesteric liquid crystals encapsulating analyte molecule.

Supramolecular receptor systems offer a sophisticated way for the gas-phase detection of low functionalised molecules that lack pronounced interaction sites. The host-cavities engulf analyte molecules in an enzyme analogous mechanism. Using cholesteric liquid crystals a similar approach can be applied for the detection of volatile organic vapours. The short range interactions within the liquid crystal (LC) molecules are of a similar nature as those which can be formed between analyte and LC. The analyte incorporation in these ordered phase cholesteric liquid crystals can be supposed, which will disturb the anisotropic phase thus changing the optical properties. Liquid crystalline behavior should not be understood as one single molecule but as the cooperation of assembled group of elements forming a supramolecular architecture. The flat, parallel molecules in a cholesteric phase are arranged in layers that, referring to the director of the molecular orientation, are twisted at a certain angle with respect to one another, so in this way, ultimately a helical structure is generated, as shown in Figure 1a. The pitch of CLCs, *i.e.*, periodicity of the helices is the feature that describes such a system. The incorporation of analyte molecule results in a structural change due to the alteration of the twist angle or elongation of the distance between the layers along the helical axes (Figure 1b). This influences the pitch height (P) and thus changes the optical properties that can be observed by recording the UV-VIS spectra of the system. In this way, the system becomes able to recognize and select different solvent molecules according to their size, which imparts selectivity to these coating materials.

**Figure 2.**
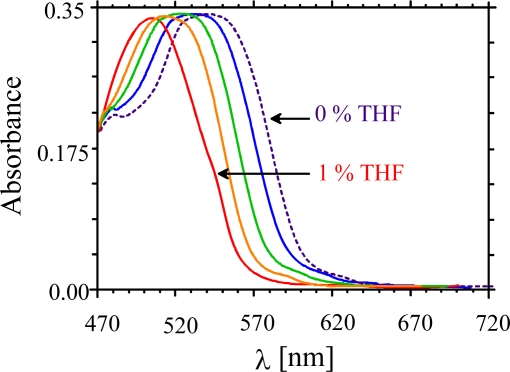
A decrease in the λ_max_ with increasing concentration of solvent vapours.

**Figure 3.**
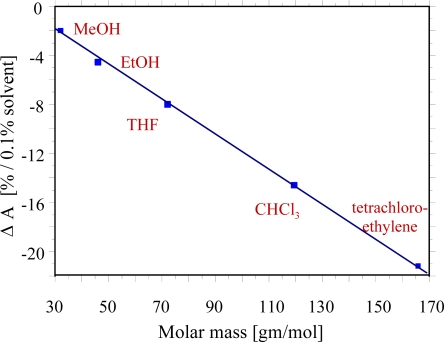
Optical sensor response of CLCs towards molar masses of different analyte vapours.

With a mixture of chiral biphenyls (Licritherm TM 1013, Merck AG) as sensitive material, analytes can be detected down to a few ppm. The dotted curve in Figure 2 shows the VIS-spectrum of a mixture with a maximum absorbance at 553 nm at 20 °C. When the LC is exposed to an increasing vapour concentration of polar or halogenated solvents a shift of the absorption band towards smaller wavelengths occurs. The effect, which is fully reversible, has been demonstrated in Figure 2 for an increasing tetrahydrofuran (THF) concentration in air up to 1.0%. The optical changes of the layers can be characterized by absorbance shifts when the wavelength of observation is fixed. The sensor characteristics were studied on various other solvents, such as methanol, ethanol and the environmental toxins such as chloroform and tetrachloroethylene, and a linear relationship between analyte concentration and absorbance is evident. The cholesteric material is able to discriminate between different solvents and offers a way for molecular shape recognition. The linear correlation between the shift of absorbance ΔA and the molar mass of the solvent as demonstrated in Figure 3. It indicates that the weight and shape of incorporated analyte vapours influence the pitch of anisotropic phase and thus changing its optical properties directly.

The bulky, halogenated analyte molecules chloroform and tetrachloroethylene seem to disturb the molecular short range order of the cholesteric liquid crystal to a larger extent, whereas polar solvents such as methanol and ethanol show only minor effects in a concentration range up to 0.5% solvent vapour in air. Going from methanol to tetrachloroethylene, less polar solvents are incorporated more effectively due to more pronounced interactions with the liquid crystalline material.

**Figure 4.**
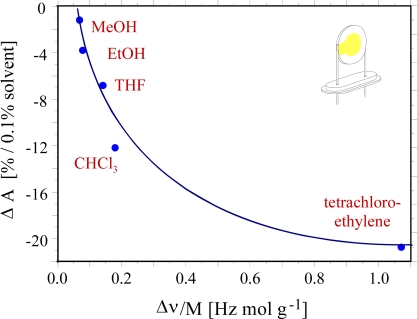
Optical absorbance changes *versus* relative QCM response for different analyte vapours.

To examine the incorporation of analytes by the sensitive layer, further mass-sensitive measurements were performed. Here the liquid crystal mixture served as sensitive coating material on a 10 MHz QMB resonator and the device was exposed to an environment with an increasing solvent vapour concentration. In Figure 4 the optical absorption as a function of change in frequency, *i.e*., mass-normalized sensor response S_mass_ = Δν/M_solvent_ is described. The term normalized response (S_mass_) is defined as ratio between frequency shift Δν at 1,000 ppm solvent concentration and the molar mass of the analyte M_solvent_ to eliminate the pure weight effect.

Comparing the optical absorption as a function of frequency shift, when going from methanol to chloroform a nearly linear correlation between optical and mass-sensitive results was observed. Here the amount of analyte incorporation is directly related to the molar mass of the analytes. This behavior changes for the bulky tetrachloroethylene molecule which is more strongly intercalated than chloroform by a factor of six. The optical change for tetrachloroethylene was not high as expected. Considering the optical measurements, the increase in the pitch height should be at the same factor larger for tetrachloroethylene but the optical sensor response is only 1.5 fold higher. Consequently the optical response has to be corrected by a molecular specific rotation parameter for tetrachloroethylene. This suggests that the tetrachloroethylene with bulky chlorine atoms will enhance the pitch of the cholesteric liquid crystals and partially will compensate for the rotation of the distinct layers to each other. Thus, the combination of mass-sensitive and optical detection principles give further insight into the sensing mechanism. In all measurements the temperature was kept constant using a thermostat. Another possibility would be to use pattern recognition strategies [20] applied for band shape analysis by multivariate detection. The increase in temperature linearly shifts the absorption band maximum to longer wavelengths. The partial least squares (PLS) method was used for band shape and band shift analysis to study the effect of temperature. In this process, eight different wavelengths in a defined range were selected taking varying concentrations of tetrachloroethylene as analyte vapours. The temperature was also varied at these different wavelengths accordingly. As a result of this analysis, a clear separation between different temperatures and varying concentrations of analyte vapours was achieved. In this way temperature can be evaluated in an independent way and a more accurate sensor response can be determined.

**Figure 5.**
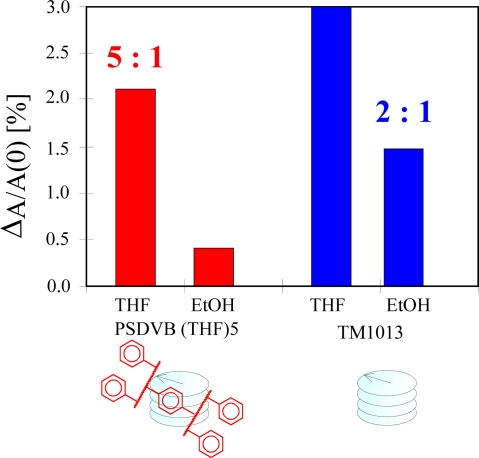
Selectivity comparison of pure and imprinted CLCs in a cross linked polymer matrix (THF imprinted) for THF and EtOH sensing indicating imprinted one is more selective [5:1] while pure CLCs has [2:1].

To improve the stability of the LC-coating against mechanical flooding, the cholesteric phase was embedded into a polystyrene matrix. In order to generate robust and porous materials that still allow analyte diffusion in the polymer, divinylbenzene was added to a styrene monomer mixture as a cross-linker in an optimized amount of 30 vol.%. Further selectivity enhancement was performed by applying the technique of molecular imprinting. The polymerization reaction was initiated by AIBN. The latter evaporates during the polymerization process due to the heating of the reaction mixture and a rigid polymer coating adapted to the analyte of interest results. CLCs in the imprinted polymer matrix provide selectivity for solvent vapour detection, which was determined through an optical transduction scheme. It has been observed that the optical absorbance for THF used as template is twice that of ethanol when pure LCs were used as sensing material, while in the case of the imprinted polymer having embedded CLCs, the absorbance of THF is about five times higher than ethanol, which demonstrates the improved selectivity provided by the imprinting matrix. The cavities formed by molecular imprinting are template oriented and favour the re-inclusion of target analyte vapours. Figure 5 shows the difference between the pure LCs and in imprinted matrix (THF) for the same solvent vapours detection indicating that the imprinted system is much more selective than the pure material. CLCs were added in optimized amount in imprinted polymer to attain enhanced selectivity.

**Figure 6.**
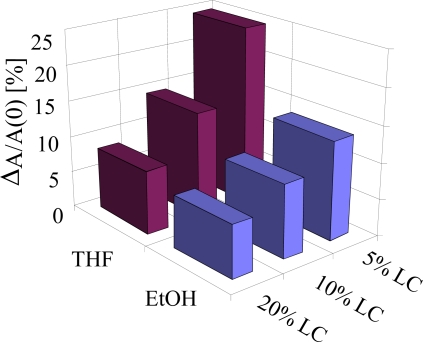
Imprinting of different amounts of CLCs embedded in cross linked polyvinyl dibenzene showing that 5% gives better selectivity than others.

This study was further extended and CLCs were added to an imprinted polymer system in different percentages, *i.e.*, 5%, 10% and 20% and cross sensitivity was observed. It has been noted that 5% of CLCs in cross-linked polystyrene matrix demonstrated the best selectivity among all for the target analyte vapours, *i.e.*, THF in comparison to ethanol. It is obvious from Figure 6 that the difference in optical absorbance of THF and ethanol is much higher for 5% inclusion of CLCs in imprinted polymer, while for 20% the absorbance is almost same for THF and ethanol. The study is quite useful for synthesizing an optimal imprinted polymer system to obtain high selectivity and reduce cross sensitivity in order to generate suitable sensor materials for hazardous solvent vapour detection.

**Figure 7.**
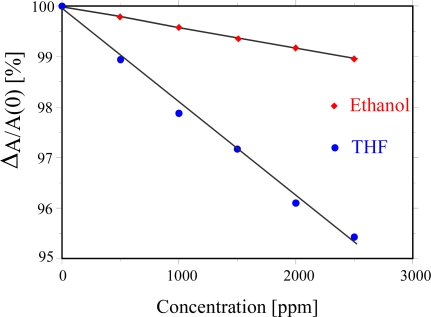
Optical response of CLCs embedded in imprinted polymer for THF and EtOH showing enhanced selectivity for imprinted THF than EtOH.

The imprinted LCs was put under examination and equal concentrations of THF and ethanol were exposed to them and the corresponding optical response was measured, as presented in Figure 7. It is evident from the graph that there is a linear sensor response for exposed THF and ethanol vapours. As a result the separation properties of the LC are effectively increased by the supporting effect of the imprinted matrix which is mass-shaped to the analyte of interest. In the measurements reported here the temperature dependence was avoided by thermostating the test gas as well as the optical cell with an integrated temperature control of the measuring equipment.

The temperature effect on LC-phases can be further separated from solvent interactions by multiple wavelength and band-shape analysis as investigated with a mixture of cholesteryl esters as sensitive material [20].

## 4. Conclusions

Due to their marked photostability, reversibility and high sensitivity, cholesteric liquid crystals offer widespread applications as sensor materials, especially for the detection of solvent molecules without pronounced reactivity. The combination of optical with mass-sensitive system provides a balance between mass adsorption and sterical intercalation in CLCs morphology which is included in the optical sensor response. Furthermore, embedding of cholesteric phases into polymer matrixes leads to tough and mechanically stable coating materials that in general show high selectivity than pure materials.
